# A Novel Approach to Sternal Fracture Repair With the Implementation of a Compression/Distraction Device

**DOI:** 10.7759/cureus.33218

**Published:** 2023-01-01

**Authors:** John Hulse, Evert Eriksson

**Affiliations:** 1 Surgery, Medical University of South Carolina, Charleston, USA

**Keywords:** surgical stabilization of sternal fracture, trauma, compression / distraction devise, open fracture reduction, sternal fracture

## Abstract

Sternal fractures are common following blunt traumatic injury. Most sternal fractures can be managed successfully nonoperatively; however, surgical fixation should be considered in certain scenarios. Specifically, surgery may be indicated in cases of severe pain, respiratory failure or dependency on mechanical ventilation, cosmetic deformity, malunion, disunion, and compression of the heart. A variety of surgical approaches to sternal fracture fixation have been documented (steel wire, suture materials, a seven-hole aluminum plate, an eight-holed Sternolock X plate, sternum-osteosynthesis plate, t-shaped plate); however, few techniques have been discussed for the initial reduction of the sternal fracture prior to fixation. In this paper, we describe a novel surgical technique used to reduce sternal fractures and approximate the edges of the sternum using a compression/distraction device.

## Introduction

Sternal fractures occur in 3%-8% of trauma patients, most commonly from blunt mechanisms [1]. Most sternal fractures can be managed successfully nonoperatively. Surgical repair of sternal fractures is not well studied in the literature, which primarily consists of case reports and case series, but there are specific scenarios where surgical repair is warranted. In particular, surgical fixation should be considered in cases of severe pain, respiratory failure or dependency on mechanical ventilation, cosmetic deformity, malunion, disunion, and compression of the heart [1-6]. A variety of surgical approaches to sternal fracture fixation have been documented (steel wire, suture materials, a seven-hole aluminum plate, an eight-holed Sternolock X plate, a sternum-osteosynthesis plate, t-shaped plate). However, only a few techniques have been discussed for the initial reduction of the sternal fracture prior to fixation [1-6]. In the following report, we describe a novel surgical technique for sternal fracture reduction using a compression/distraction device.

This article was presented as a poster presentation at the Chest Wall Injury Society Summit in April 2022.

## Case presentation

Case one

**Figure 1:**
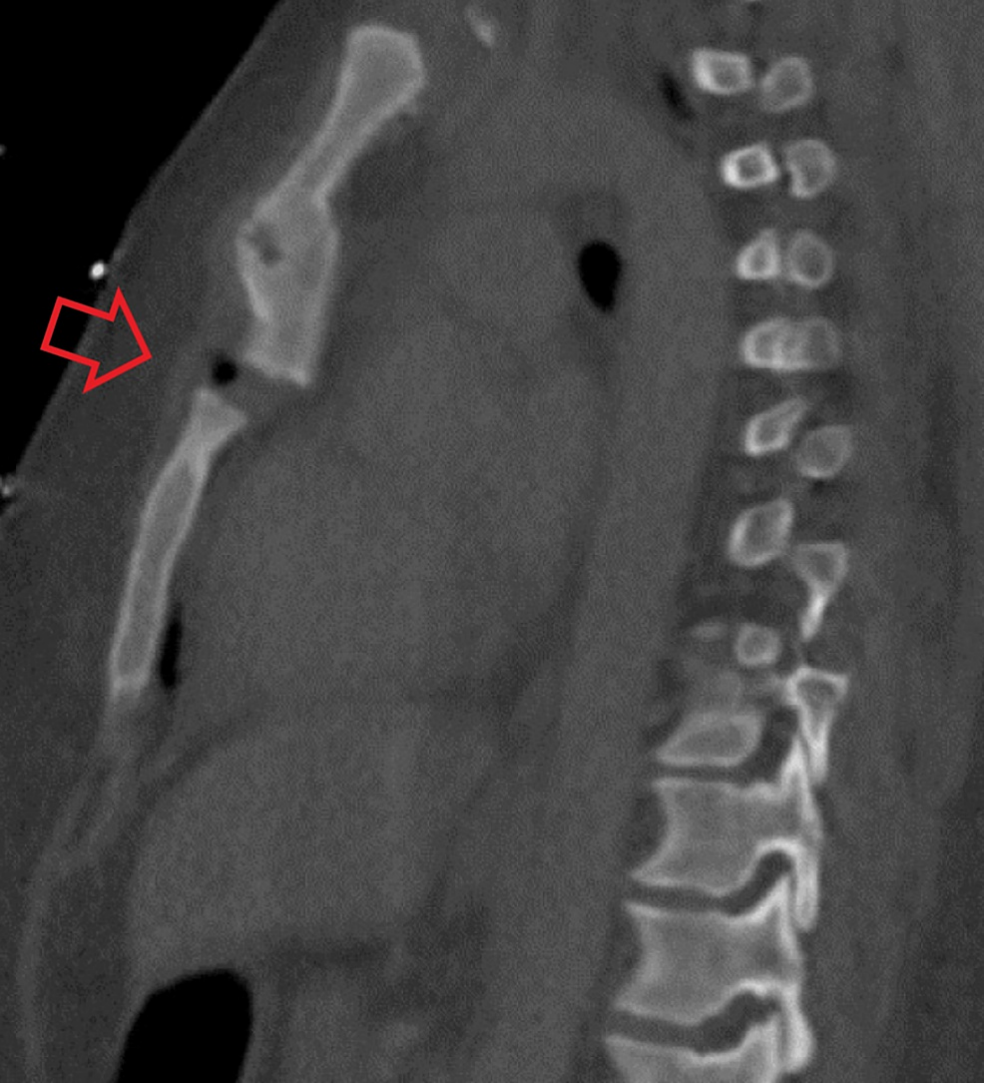
Sagittal CT image demonstrating posteriorly displaced and superiorly distracted sternal fracture (bone window). The red arrow points to the location of the fractured sternum.

**Figure 2:**
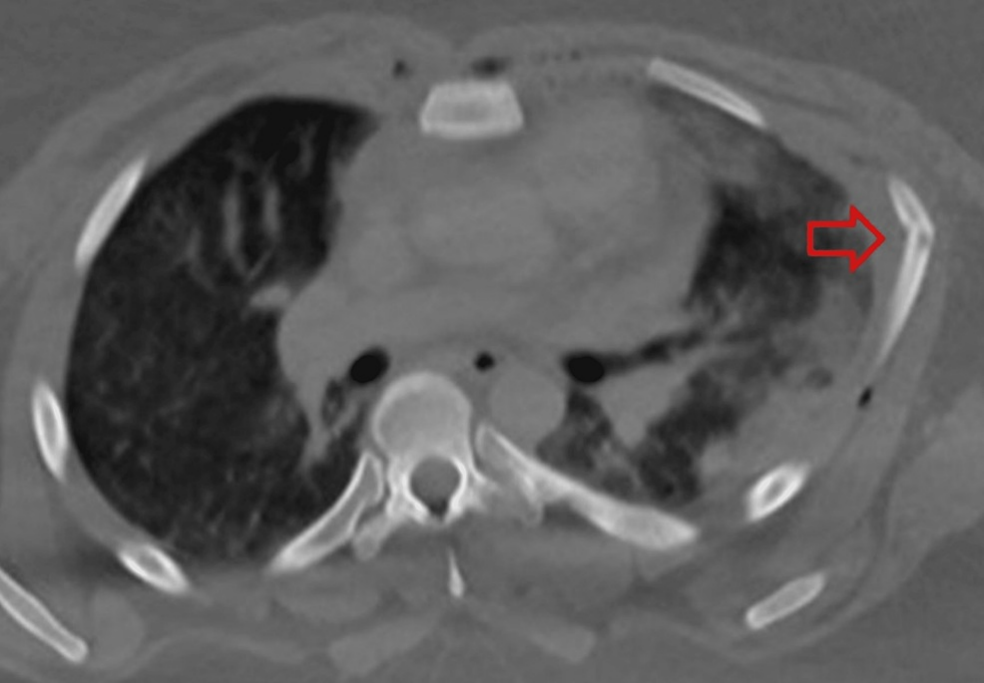
Example of anterior lateral left-sided rib fractures noted by the arrow.

**Figure 3:**
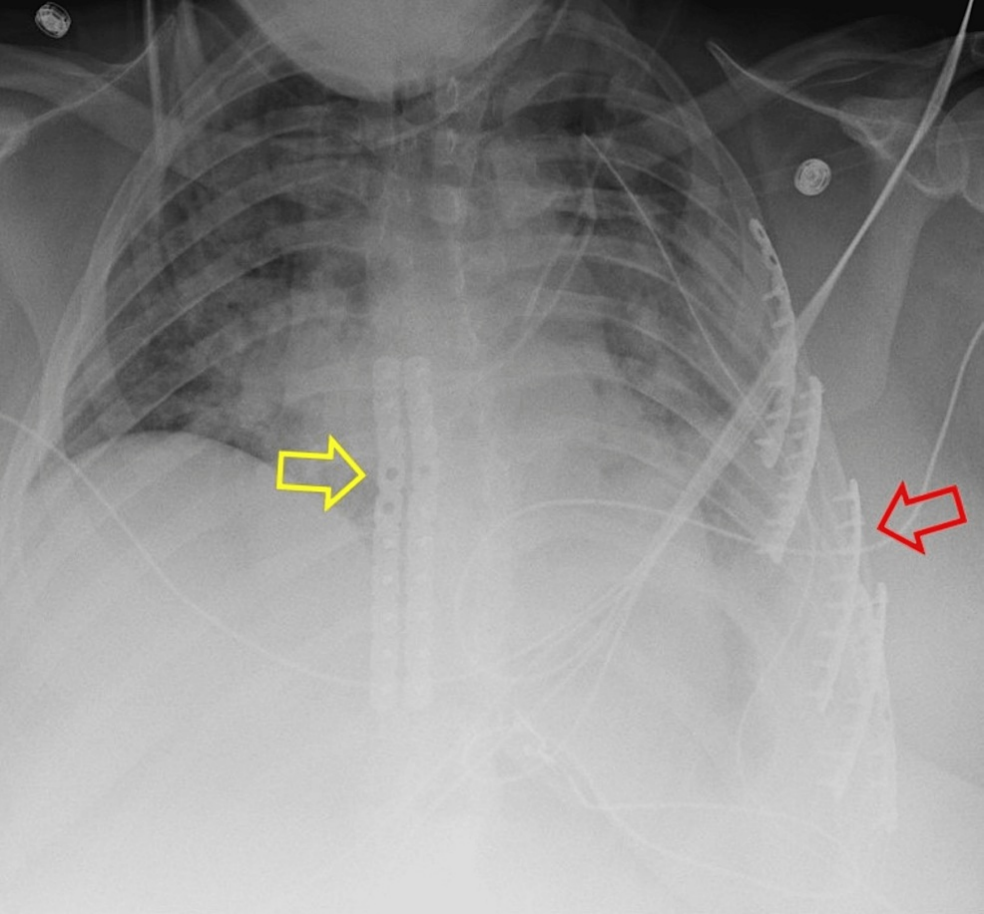
Post-operative chest x-ray. The yellow arrow indicates the sternal plates, and the red arrow indicates the rib plates.

A 29-year-old female that was involved in a motor vehicle collision in which she was unrestrained and ejected. She suffered polytrauma including left-sided displaced anterolateral rib fractures (Ribs 2-7) as well as an acute-on-chronic sternal fracture. The chronic sternal fracture was from a motor vehicle collision when she was 18 years old and had caused her pain since the injury. On hospital day 1, She developed respiratory failure in the intensive care unit and had paradoxical chest wall motion on the ventilator. The patient was unable to be weaned from the ventilator due to pain from the rib and sternal fractures and therefore rib and sternal fixation were undertaken on hospital day 3. A representative image from the computed tomography (CT) acquired preoperatively of the sternum is shown in Figure 1 and the rib fractures are in Figure 2. Two plates were placed on the sternum as well as plates on ribs 3-7. A postoperative chest x-ray can be seen in Figure 3.

Case two

**Figure 4:**
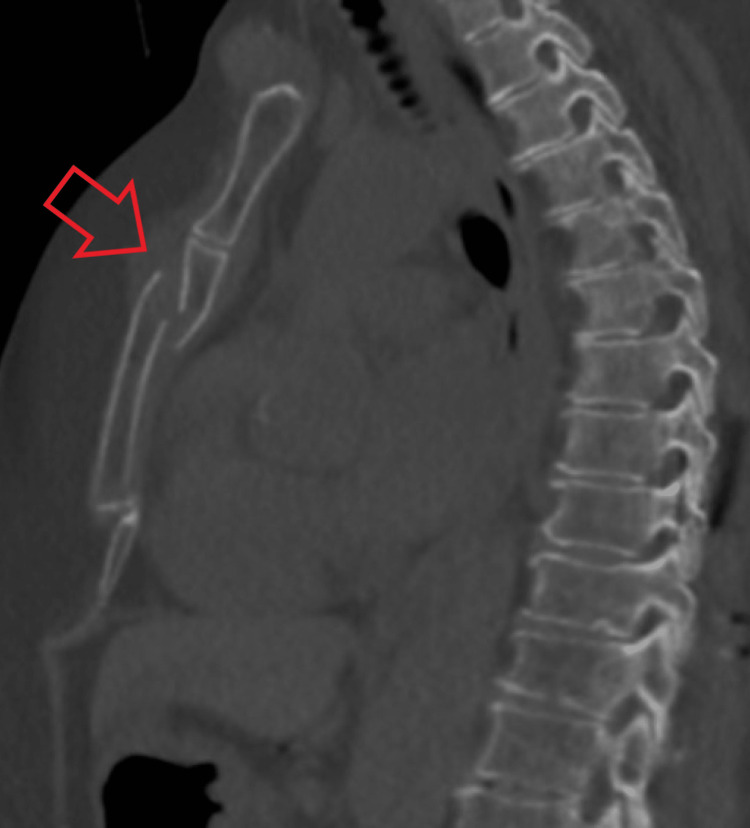
Sagittal CT image demonstrating posteriorly displaced and inferiorly distracted sternal fracture (bone window). The red arrow indicates the fractured sternum.

A 64-year-old female suffered a ground-level fall resulting in a posteriorly displaced bicortical fracture of the body of the sternum. The patient developed severe pain and respiratory insufficiency requiring open reduction and internal fixation. A representative image from the CT acquired preoperatively is shown in Figure 4.

Surgical technique

In case one, the rib fractures were addressed first. A left-sided thoracotomy was performed followed by a five-level open reduction internal fixation of the fractured ribs. Case two did not require surgical repair of rib fractures.

Exposure

Both cases underwent a similar approach to sternal fracture repair. The patient was placed in a supine position, and the anterior chest was prepped and draped. A vertical incision was made overlying the sternum and fracture. Electrocautery dissection was used to dissect down to the midline of the sternum. The fracture could be easily palpated at this stage. The insertion of the pectoralis major on the right and the left side was elevated to the edge of the sternum using electrocautery. In case one, the acute on chronic nonunion fracture was evaluated and a pseudoarthrosis cavity was present and debrided sharply. The superior and inferior aspects of the bone were debrided using a 4.0-mm oval solid carbide tip burr (Product Number 5300-020-901, Stryker Instruments, Kalamazoo, MI) back to normal bone. The intramedullary canal on the superior and inferior aspects was exposed.

*Distraction and Compressio*n

**Figure 5:**
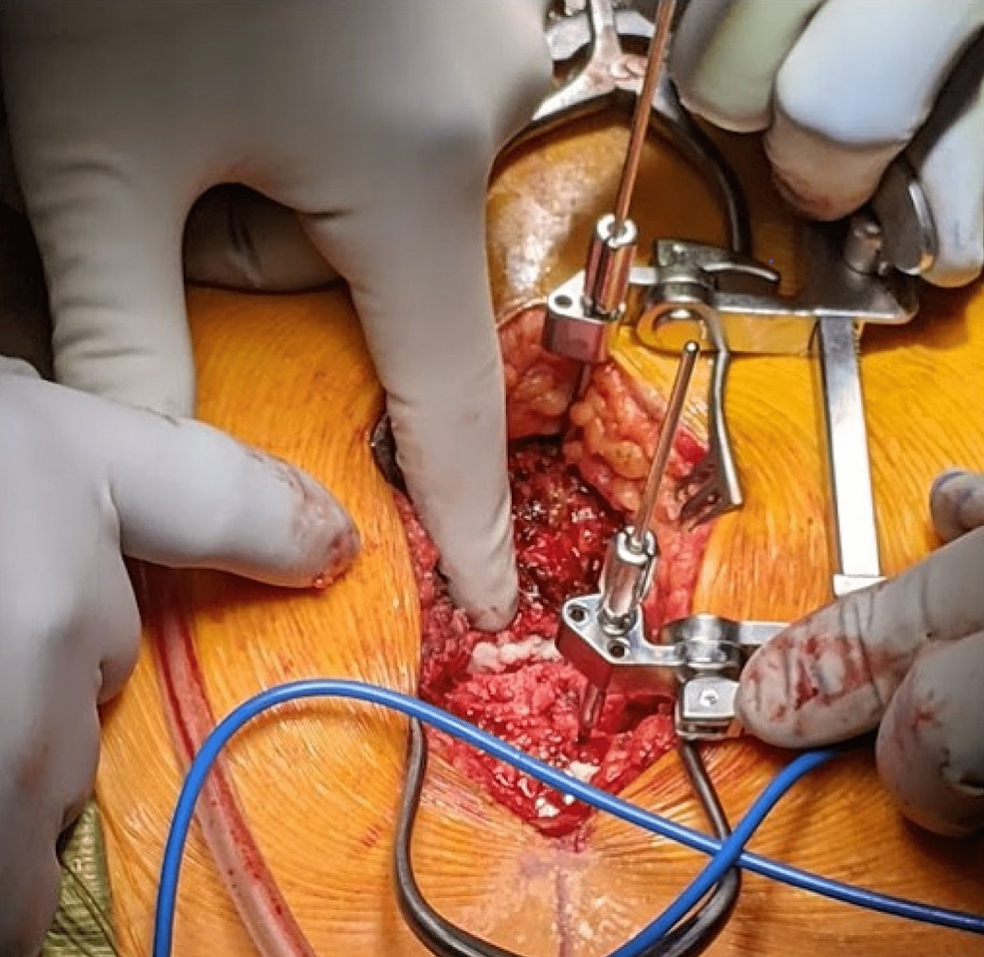
Intraoperative image with compression/distraction device in place just following distraction of the sternal fracture.

A compression/distraction device (Device number 01.211.002, Depuy Synthes, West Chester, Pennsylvania) was applied. The threaded Kirshner wires (k-Wire) (2.5 mm diameter) were placed into the sternal body superior and inferior to the fracture. Meticulous control of the insertion of the k-wire was necessary to not penetrate the posterior sternal body. The thickness of the sternum was measured on the preoperative CT scan and the appropriate thickness was marked on the k-wires. Monitoring this mark on the k-wire allowed insertion without penetrating the posterior sternal cortex. This is of paramount importance as the heart and lungs sit just deep into the sternal body and injury could be life-threatening. The device was first used to distract the bone segments to eliminate any posterior displacement and restore alignment. Next, it was used to reduce the bone segments into compression in their normal anatomic orientation. In case one, the compression/distraction device was able to eliminate a 1-cm defect that was present from the acute on chronic nonunion fracture. In case two, it was used to elevate and elongate the sternum from its retrosternal position by greater than 1 cm. Figure 5 shows the positioning of the k-wires as well as the compression/distraction device in position allowing the reduction of the fracture.

Fixation

**Figure 6:**
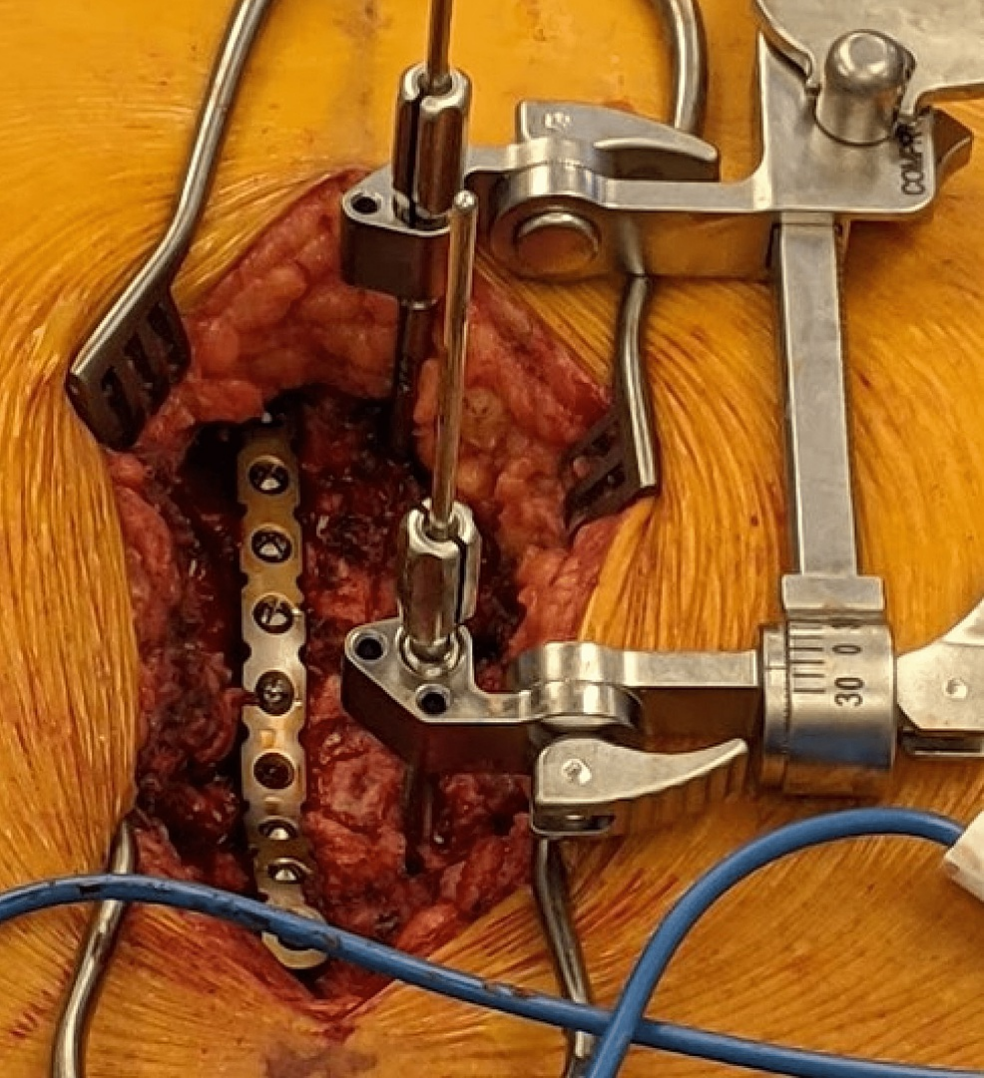
Intraoperative image with the compression/distraction device in place with a sternal plate in place with bicortical screw fixation.

A sternal plate was custom bent to the contour of the sternum. Bicortical fixation screws were placed with three above the fracture and five below the fracture. The depth of the sternum was measured preoperatively on the CT scan, which determined the length of screws used. Figure 6 shows the sternum being held in position by the compression/distraction device and a vertical titanium plate and bicortical screws holding it in position. Prior to placing the second plate, the compression/distraction device was removed from the sternum. A second sternal plate was custom bent to the contour of the sternum and secured parallel to the first plate in a similar manner with three screws above the fracture and five below the fracture. The sternum was repaired and held in reduction with compression of the fracture.

Closure

Hemostasis was obtained and the wound was then closed in layers to reapproximate the pectoralis major fascia/muscle, Scarpa's layer, deep dermal layer, and the skin.

## Discussion

These two cases illustrate presents a novel surgical technique used to reduce sternal fractures and approximate the edges of the sternum using a compression/distraction device. Little has been published on techniques for sternal fracture reduction to reapproximate the fractured segments and provide compression across the fracture line. The articles cited in this report either briefly state that the fracture was reduced using bone clamps or reduction forceps [1-3,6] or make no mention of the technique used for fracture reduction [4,5].

Reducing the sternal fragments to their anatomic position usually necessitates an approach to the posterior wall of the sternum or placing elevators into the fracture lines to pry the bone fragments back into normal anatomic position. The current technique used by most surgeons involves the use of bone clamps or reduction forceps introduced laterally into the surrounding intercostal spaces to reduce the sternal fracture. This technique may injure mediastinal organs or surrounding vessels, most notably the internal thoracic vessels and the sternal blood supply [1,6]. Other techniques to elevate the sternum require placing a freer/elevator into the fracture line to bring the posteriorly displaced bone up into alignment [1]. This does not allow compression of the fractured sternum and may result in a gap between the fractured pieces.

Using the compression/distraction device technique described above reduces the amount of dissection posterior and lateral to the sternum thus reducing the risk to the surrounding structures. Another main advantage to using the compression/distraction device is the control the crankshaft provides in distracting and then reducing the fracture when compared to manually manipulating the bone segments with forceps or clamps. Finally, the compression/distraction device can easily hold the bone segments firmly in place as well as apply compression while preparing for sternal fixation with plates.

It is worth acknowledging the limitations to the implementation of the compression/distraction device to sternal fracture reduction. Most notably, the depth that the K-wire is inserted must be carefully calculated and performed. There is a real risk of injury to mediastinal structures if the K-wire is inserted too deep or if a malfunction in the drill results in the wire being placed too deep. Another current limitation is that it has only been applied to transverse sternal fractures. Its application has not been attempted for longitudinal or diagonal sternal fractures or comminuted fractures with multiple sternal segments. The last limitation is the availability of compression/distraction devices in some hospitals. Fortunately, many hospitals will already have this device available because it is commonly used in Orthopedics as an “ankle distractor.”

## Conclusions

In summary, we present a novel approach to the reduction of sternal fractures prior to sternal fixation using a compression/distraction device. This method has been applied successfully to multiple transverse sternal fractures. This method of reduction avoids the more lateral and posterior dissection along the sternum required by some reduction techniques. Additionally, the compression/distraction device gives superior manual control over distraction and then compression of the sternum. We believe the technique presented here is a more reliable and potentially safer method of aligning displaced sternal fractures.
